# Complete genome sequence of the first *Capnocytophaga canimorus* strain isolated from a red fox (*Vulpes vulpes*) in Germany

**DOI:** 10.1128/mra.01228-24

**Published:** 2025-01-13

**Authors:** Peter Kuhnert, Isabelle Brodard, Joerg Jores

**Affiliations:** 1 Institute of Veterinary Bacteriology, Vetsuisse Faculty, University of Bern, Bern, Switzerland; Rochester Institute of Technology, Rochester, New York, USA

**Keywords:** mesocarnivore, zoonosis

## Abstract

The zoonotic pathogen *Capnocytophaga canimorsus* is part of the oral microbiome of dogs and cats. We report the genome sequence of strain KM3195_24, isolated from a red fox. It has a circular genome of 2,718,701 bp with a G + C content of 36%, 2,419 CDS, 3 rRNA operons, and 45 tRNAs.

## ANNOUNCEMENT

*Capnocytophaga canimorsus* is part of the canine and feline oral microbiome. It has also been previously reported as zoonotic pathogen in immunocompromised humans causing generalized infections mainly after animal bites (1, 2). Only a small fraction of serotypes found in cats and dogs have been implicated in human infections (3). *C. canimorsus* has not been reported in wild mesocarnivores.

Strain *C. canimorsus* KM3195_24 was isolated from the oral cavity of a juvenile red fox (*Vulpes vulpes*). The animal was legally hunted in the state of Mecklenburg-Vorpommern in Germany in August 2024 by the last author holding the necessary hunting license. The oral cavity was swabbed using the Opti-Swab Liquid Amies collection & transport system (Puritan), transported to the laboratory at ambient temperature, and plated on Brain Heart Infusion agar (BHI; Oxoid) containing 5% sheep blood (Thermo Fisher Scientific) and 20 µg/mL gentamicin. After microaerophilic growth at 37°C for 3 days, a single colony was picked and subcultured on Fastidious Anaerob Agar (FAA; Neogen) containing 5% sheep blood and 5% rabbit serum (Gibco). Initial identification was performed using matrix-assisted laser desorption ionization-time of flight (MALDI-TOF) mass spectrometry (Microflex LT/SH; Bruker). The isolated strain designated KM3295_24 was stored at −70°C.

Genomic DNA was isolated from fresh-grown KM3295_24 on supplemented FAA plates according to (4) using phenol:chloroform:isoamyl alcohol (25:24:1) for extraction, followed by additional purification with the DNeasy PowerClean Cleanup Kit (Qiagen).

Sequencing was done using both Oxford Nanopore Technology (ONT) and Illumina sequencing at Microsynth AG (Balgach, Switzerland). Libraries were prepared using the Rapid Barcoding Kit 96 (SQK-RBK114.96; ONT) and DNA Tagmentation using unique dual indexes according to the manufacturers’ instructions (Illumina), respectively. The ONT libraries were sequenced on a PromethION 2 on a FLO-PRO114M flow cell and the Illumina libraries on a NovaSeq6000 apparatus using an SP sequencing kit with 500 cycles in paired-end fashion.

ONT sequencing revealed a coverage of 84× with a total number of raw reads of 27,676 (N_50_ 21,182). The software dorado (version 7.3.11) was used for base calling, demultiplexing, and adaptor trimming. Read quality was checked using NanoPlot (version 1.42.0) and then quality filtered using filtlong (version 0.2.1), discarding any long-reads with an average quality below Q10 and a read length below 1,000 bp. Finally, assembly was done using flye (version 2.9.3). The resulting contig was polished by applying racon (version 1.5.0) and medaka (version 1.12.1). Illumina sequencing revealed a coverage of 780× (4,238,968 raw reads; 250 bp, paired end). Illumina reads were quality-filtered and trimmed using bbduk (version 38.96) and then used in combination with pilon (version 1.24) and polypolish (version 0.6.0) to further polish the assembled contig. The genome was rotated to the first nucleotide of the start codon of the *dnaA* gene. The annotation of the closed and circularized genome was achieved using PGAP (version 2024-07-18.build7555). Default parameters were used except where otherwise noted.

The genome consists of 2,718,701 bp with a G + C content of 36%. It harbors 2,419 CDS, 3 rRNA operons, and 45 tRNAs.

## Data Availability

The genome sequence has been deposited in GenBank under accession number CP173182 and project number PRJNA1182120. The raw reads from Illumina and ONT sequencing were deposited under accession numbers SRR31278853 and SRR31278854, respectively.
